# Monthly Summary

**Published:** 1868-01

**Authors:** 


### MONTHLY SUMMARY.

---

*Food for Babies.*—From an interesting article on "Food for Babies," published in the London *Medical Times and Gazette*, we make the following extracts.—*Boston Med. and Surg. Journal.*

Of milk, we have that of the ass, goat, and cow. Asses' milk is by general consent the best substitute for the woman's for most delicate children; and, dear as it is, it is well worth the money. The goat's is a rich milk, and with a strong curd, and only adapted for robust children. The milk of the cow is, of course, the staple. And whilst for general purposes it is quite right that milk should be brought from any distant part of the country, it must be confessed that a few cows should be kept in town in hot weather, that their milk may reach the baby part of the population fresh, unshaken, and just as yielded by the animal. But cow's milk is too rich in curd for the human baby, whose muscular movements are almost confined to breathing, crying, and the heart's action, So it must be thinned, and the simplest way is the common one of adding an equal part of water (the water being gradually diminished as the child grows older) and a small quantity of white sugar. It is a refinement to use the sugar of milk, instead of common cane sugar, but whether there is anything gained we never could satisfy ourselves.

The test of any kind of baby's food is found in the fact that the child thrives—that it is satisfied after its meals, not subject to fits of pain in the stomach and flatulence, nor yet to fits of colic in the bowels—and that the residum, which is generally produced upon a napkin for inspection, does not show undigested food. All these things are self-evident. A child ought regularly to grow, to be plump, and to gain in weight every week, and if it do not, something is wrong. Secondly, the child ought to be satisfied and go to sleep after its food ; but here the junior practitioner ought to be aware of one physiological fact—when a child is in pain in the abdominal organs, it often displays insatiable hunger, has a tendency to suck greedily, and this though the stomach and bowels may loaded with undigested food. Ignorant nurses kill many a child by inattention to this point. The child cries after food ; therefore they say the food is not good enough, "the milk does not satisfy," &c., and forthwith they give the child some half-solid pap, and dose the mother with over-rich food and alcohol. A purgative dose of oil is the best remedy when a baby is unreasonably hungry after food ; castor oil is generally used, but any oil or soft fat will answer the purpose. The old custom of giving a bit of the fat of a pig is founded on reason and experience. Lastly, the practical fact remains that no undigested food ought to be found in a baby's napkin. Any mother may be taught that lumps of curd and masses of undigested starch can give the child no nourishment, but decompose in the bowels, and cause first pain, next diarrhœa. A healthy baby's napkin should not be offensive—of course, it has a faint peculiar odor, but certainly it does not stink, and it do, either improper food has been given, or proper food has not been digested.

In other cases, in order to diminish the proportion of curd, it is useful to give *cream* diluted with new milk and water ; and, to prevent the curd of cows' milk from coalescing into hard lumps in the stomach and passing undigested, the milk may not only be diluted with water, but with effervescing soda-water (this is called artificial asses' milk) or potass-water or lime-water. Sometimes a very little of the solution of magnesia is added.

But this purpose (*i. e.*, the making the curd softer and more digestible) is generally effected by mixing it with cereal food or the starches. Theoretically speaking, we do not want the nitrogenous elements of the cereals, because the cow's milk contains enough of them. Hence, arrowroot or sago may suffice, if it be understood that the child is to live upon the milk, and that these starchy elements are superadded to modify the milk and not to be substitutes for it. Still, general experience is in favor of some cereal. Barley-water made from pearl barley, and mixed with an equal part of milk, is an admirable food for most children. Robinson's patent barley deserves praise. Oatmeal gruel and milk agrees well with the robust. Brown and Polson's preparation of maize and the maizena, seem favorite preparations. On the whole, however, wheat tends to displace the other cereals. The flour of wheat is often baked or broiled, and when so cooked is boiled afresh with water and milk. Or it is made into biscuits, of which Robb's, Lemann's, the Norwich knobs, "tops and bottoms," and rusks, are popular samples ; or into a farnaceous food —that is, a powder composed of wheat flour or biscuit, with or without admixture of other cereals, and already acted on by heat, so as to require little or no cooking (Hard's, Neave's, &c., &c.)

This is the place to notice "Liebig's soup," a compound of milk, wheaten flour, and malt, with a small quantity of bicarbonate of potass The object of the malt is to convert the starch of the wheat into sugar, and so to save the stomach the trouble of that process : whilst the cow's milk is enriched with the phosphates of the wheat and the added alkali. The thanks of society at large are due to Liebig, not only for the care and patience with which he has worked this idea out, and the liberality with which he published it, but likewise for the impetus which it has given to the study of the whole subject of infant food in connection with mortality.

The original recipe prescribes ½ ounce of wheaten flour, ½ ounce of ground malt, and 7½ grains of bicarbonate of potash, to be well mixed with 1 ounce of water; then 5 ounces of cows' milk are added, the whole is heated gently till it thickens; then it is removed from the fire, stirred till the starch is converted into sugar, as indicated by the liquid becoming thin, again boiled and stirred for some minutes, and lastly strained. For use, this requires to be much diluted for young babies, less for older ones. * * * * * * * * * *

As for results. We believe that of any six infants one would refuse to swallow it; one would take it without benefit: but but that the remaining two-thirds would take it greedily and thrive on it. We have known it put a stop to so many of the miseries arising from undigested or indigestible food, that it has, we think, already earned for itself a permanent place. What form of it will ultimately be the favorite is another question.

The objections to Liebig's food in its common form are, first, the time, trouble and nicety—it cannot be prepared in less than twenty minutes, and not every nursemaid or mother has the intelligence sufficient. Secondly, there is the considerable amount of indigestible husk, often very difficult to separate by straining, and consisting of spicula that look very formidable to any tender mucous membrane, Thirdly, as a theoretical objection, we mention its too saccharine nature and the absence of fat.

The first objection has been met by Savory and Moore, who have put together and prepared the ingredients in such a way that they only need the addition of water and milk, and no straining nor boiling. Mr. Mellin's preparation, if it can be got, of course avoids all trouble of cooking; and we may say that the malt he uses is most scrupuously cleansed from husk. There is also to be procured at Mr. Van Abbott's a preparation called "Liebig's Food for Infants concentrated," the invention of Mr. Ed. Lœflund, chemist, of Stuttgart; it is a thick syrup, containing a concentrated solution of the wheat and malt elements. It has, when mixed with milk in due proportion, a sweet, somewhat empyreumatic, bitter taste, and this is the general character of the food, however prepared; but there is a distinct acid tracely reaction in Mr. Lœflund's syrup. Mr. Mellin has made an extract in the form of granular powder, soluble in cold water, very palatable, free from acidity, and much more portable than Lœflund's syrup. Lastly, we must notice the very ingenious malt biscuits made by Spiking, of Dover Street; these contain the malt and wheaten flour in the form of a biscuit; of course they are portable, and keep any time, and require no more cooking than Robb's or any other nursery biscuit. * * *

We have now, we trust, set forth a pretty general view of infant's food, and shall add but three or four practical hints:—1. The advantage of adding cream from time to time, especially if the baby is constipated. Want of fat is the cardinal defect in Liebig's soup. 2. The expediency of adding a small quantity of some aromatic water to all infants' food, such as dill, anise, &c. There is a very popular food in some countries, consisting of equal parts of barley-water and milk, with one teaspoonful of good brandy to the pint. Bad for the babies' livers, some would say; but no harm is found in practice. 3. The expediency of giving delicate children small quantities of pure gravy or beef-tea, sweetened or a few grains of raw meat ground to a pulp. If these agree, a child is almost safe. 4. No one kind of food can agree with all children. It has provoked us to see children dying on a diet which did not suit them, without an effort to shift and combine various elements till the right thing could be found. 5. The importance of teaching the poor that food for babies should be *thin*, and that a thin food may be more nutritious than a thick one.

---

*Prevention of Sickness from Chloroform.*—A writer in the *British Medical Journal*, says:

"Vomiting is so frequent and so troublesome a concomitant of the administration of chloroform both during and after the inhalation of the anæsthetic, that I am pleased to lay before your readers the general result of a very simple, and as I believe, a very effective mode of prevention. I have already, in some eighteen or twenty eye operations, adopted the plan of giving the patient a drink of a few drops of chloroform in water before commencing the inhalation, and so far the result has been most satisfactory; not more than one, or at most two, cases of slight nausea having occurred where the chloroform drink had been previously administered. The remedy has, of course, to be more extensively tested before it can be relied upon; but I should be glad if some of your correspondents would record the result of their experience in its use."

---

*Heart's action after Death.*—M. Marcelin Duval, director of the Brest Naval Medical School, has published a paper on the Physiology of the Circulatory Functions, in which he records the results of experiments made upon guillotined criminals. From a translation of an extract from this report, we gather the following interesting facts.

Five or six minutes after decapitation the carotids continued to pulsate, thrusting themselves beyond the level of the section of the neck, and then retracting, and emitting at each pulse a little frothy blood. Seven minutes after death, the chest was rapidly opened, and the heart was beating at the rate of forty-eight to the minute. The left auricle rested on the aorta, embracing it. After a short rest, this appendix rises suddenly, leaving the aorta, swelling, elongating and throwing the fringes on its edge into denticulated folds, and then falling back again upon the great artery. In one subject the heart continued to beat for an hour and a quarter, its pulsations being uninterrupted by the removal of the stomach, the intestines, the diaphragm and the lungs. The ventricles contracted simultaneously, shortening all their diameters and gathering their surfaces into folds and wrinkles, the contractions commencing at the base and running down to the apex of the organ.

---

*Splanchnoscopy.*—All the dark recesses of the human body have been pried into. Specula for the uterus, for the anus, for the ear are antiquated affairs. We peer into the larynx now, and the endoscope reveals to us the inside of the bladder. We are literally being turned inside out by modern contrivances. The latest invention is that of an ingenious Frenchman who having, in his childhood, seen his venerable grandam peer through an egg against the light to test its freshness, has conceived the bright idea of treating men and women after the same fashion. He makes his luckless patients swallow glass tubes which contain platinum-wires arranged to produce the electric light. He then connects them with a powerful galvanic battery, and a brilliant illumination of the stomach is the result. He expects in this way to render the abdominal parietes sufficiently translucent to detect tumours, indurations and ulcerations. If we could only print the significant national shrug, we should say in his own language, "peut etre."

---

*A New Micrometer.*—Dr. Van Gieson publishes in the *Medical and Surgical Reporter*, a plan of a new micrometer, which promises to be cheap, accurate and effective. It is simply an application of micro-photography. An object is prepared consisting of crossed lines at equal distances and then photographed on glass. A very simple calculation gives the absolute size of the minute squares. To preserve these lines permanently, it is only necessary to heat the glass just to the point of fusion, when they become incorporated with the substance of the glass. Of course if too great heat be used they will be warped out of shape, if too little, they will not be fused into the glass. The present high price of micrometer slides produced by machine-ruling, will, we suppose, render the profession eager to try the very simple and inexpensive process thus suggested. The value of microscopic observations, as all know, is greatly increased by the measurements which the micrometer enables the observer to make.

---

*Testing Glycerine.*—The effect of glycerine on tender and broken skin should be mild, but physicians often hear complaints of its burning and inflammatory action, even when it has been largely diluted with water. The effect of such glycerine is now known to be due to the presence of a certain amount of oxalates and formates, also traces of ammonia. Litmus paper will remain unchanged in such impure glycerine; but when the latter is mixed, in a test-tube, with its equal bulk of sulphuric acid, a strong effervescence will take place. It has also been ascertained, that such glycerine was not obtained by the process of distillation, but was only partially purified by chemical action.—*Med. and Surg. Reporter*

